# Meddlesome Midwifery

**Published:** 1871-11

**Authors:** Francis H. Milligan

**Affiliations:** Wabash, Minnesota. (Northwestern Medical and Surgical Journal)


From the Northwestern Medical and Surgical Journal.

### MEDDLESOME MIDWIFERY.

BY FRANCIS H. MILLIGAN, M.D., WABASH, MINNESOTA.

In the July number, 1870, of the "American Journal of Medical Sciences," my friend, Professor Wallace, of the Jefferson Medical College, publishes an interesting article "On the Proper Use of the Obstetric Forceps."

That there is a prejudice against instrumental delivery, and that that prejudice has existed to a very great extent in our rural districts, I apprehend will not be denied. In our cities, where consultations are more frequent than they are or can be in the country districts, the prejudice does not exist. Still, what has been termed "Meddlesome Midwifery," in town or country, is too often condemned, not only by the profession, but by the community at large.

I very well remember how "good old Prof. C. D. Meigs" used to caution his class about the use of forceps, and how he used to instill in our minds that, "to prevent the abuse," we should never carry forceps with us. Meigs was the students' favorite, and for the first ten years of my professional career, it was my habit not to carry my obstetrical case.

I saw that I erred. To illustrate: In the summer of 1860, I was called fifteen miles in the country, to see a lady said to be in labor. After a tedious journey of four hours, the roads being in a miserable condition, I arrived at the house of my patient, and found that she had been dead some fifteen minutes, the head of the child pressing on the perinæum. I had delivered the same lady two years before, with no difficulty, the labor being perfectly natural. Two physicians were present. The lady had been in labor sixty hours. A midwife was called first, who only requested medical aid when all the natural efforts were exhausted. The professional gentlemen were called the day before the lady died. The head being in identically the same position as it was at her death, neither of the medical gentlemen had the manliness or intelligence to suggest delivery by instruments. No hæmorrhage had occurred, but the case had died of sheer exhaustion and nervous excitement. Had the physicians had forceps and known how to use them, both mother and child would probably have been saved. From this occurrence, up to the present hour, when called direct from home to a labor case in the country, or any distance from my residence, I always take my instruments; and have yet to regret doing so.

The above case is only one of several which I might relate, coming under my notice, since my residence in this State. Midwives and quack doctors have a holy horror of the use of instruments in labor. Why? Midwives as a class, with a few honorable exceptions, are only ignorant old crones, with as little knowledge of their art as a four-year-old child; and quack doctors oppose manual or instrumental delivery because they are aware of their own ignorance, and hence take great pride in abusing physicians who are versed in the science of obstetrics.

Prof. Wallace remarks in his article, to which I have referred, "I deny the right of any man to attend a case of labor unless he carries his forceps with him; and I consider that teaching to be erroneous which says, go without your forceps, and send for them when you see the probable necessity of their use.

"I appeal to the experience of obstetricians who have sent for their forceps under emergency, have they not sometimes regretted that they had them not at hand for instant use in certain of these emergencies?"

Dr. Wallace further remarks that Dr. Penrose, Professor of Obstetrics in the University of Pennsylvania, tells me that he always carries his forceps with him in labor cases, and has yet to regret having applied them too often or too soon.

This, I think, is very good authority, and if the practice be necessary in large cities, like Philadelphia, it is surely so in country practice.

It may be said, however, that if we have our instruments at hand we might apply them when nature could perform the delivery. Granted; but I assert, that the physician who is so ignorant as not to know when nature fails in her work, and when art should be applied, has no business to be present at a case of labor, and probably would not have intelligence enough to know how to apply the instruments.

I claim that a woman in the pains of labor, is deserving, and should receive *all* our attention, and should not be left one moment longer to suffer than the natural efforts of nature require. However *fashionable* it might have been twenty years ago, for a medical attendant to wait for days, in labor cases, for nature to perform her work, at the present time, aided by anæsthetics, it is our duty to assist nature by instrumental and manual delivery.

This assistance, in years past, has been called "Meddlesome Midwifery;" now, I apprehend, the proper name should be, Scientific Obstetrics.

Very much of the sin of abortion among the higher classes of our American females, I attribute to the dread of the pains of labor. If anæsthetics were more generally used, as advised by the lamented Simpson, child murder among the American women, would, I apprehend, be less frequent.

I do not wish to be understood to advocate, in this paper, that the obstetrician should apply the forceps in *all* cases of labor; still less do I advocate that he should "turn and deliver" every case which is tardy in engaging. I do insist, that it should be our duty to assist and aid nature, and in every way possible to lessen the "pangs of labor," by anæsthetics and other means at our disposal.

He who fears public gossip so much as not to use instruments or manual assistance, when nature has failed, is unworthy of the name of a physician, and unfit for his calling.

In conclusion, I will here remark, that it is not the use, but the abuse, of manual assistance in labor that we should condemn. I am free to admit, that, as I grow older and more experienced, I "meddle" more to shorten the duration of labor, than I did in the early part of my professional career, with better results, both to mother and child.

